# Temporal modulation brings metamaterials into new era

**DOI:** 10.1038/s41377-022-00870-0

**Published:** 2022-06-07

**Authors:** Luqi Yuan, Shanhui Fan

**Affiliations:** 1 State Key Laboratory of Advanced Optical Communication Systems and Networks, School of Physics and Astronomy, Shanghai Jiao Tong University, 200240 Shanghai, China; 2 Department of Electrical Engineering, Stanford University, Stanford, CA 94305 USA

**Keywords:** Optics and photonics, Optical physics

## Abstract

Temporal modulations in photonics bring many exotic optical phenomena in the time dimension while metamaterials provide powerful ways in manipulating light in the spatial domain. The authors envision the connection, Floquet Metamaterials, may deliver novel opportunities in nanophotonics.

Temporal modulations on photonic materials attract great attentions in past decades, where the time-reversal symmetry can be broken and therefore exotic non-reciprocal light control has been realized^1^. For instance, periodic dynamic modulation of the refractive index makes it possible to create an effective magnetic field^2^. Such a magnetic field does not exist in nature for photons as well as other neutral wave particles such as phonons. And yet achieving such effective magnetic field is essential in the realization of quantum Hall effect for photons^3^. In general, a structure undergoes periodic dynamic modulation can be theoretically studied using the framework of Floquet analysis^4,5^. Such an analysis has led to the discovery or demonstration of many optical phenomena with fundamental physical interest, including but not limited to the Floquet topological insulator^6^, Floquet solitons^7^, time-periodic corner states^8^, and the Floquet band structure beyond the rotating-wave approximation^9^. The Floquet analysis has highlighted novel wave dynamics as enabled by controlling the temporal degrees of freedom in electromagnetic systems as governed by the Maxwell’s equations.

Optical waves can also be controlled by engineering the spatial degree of freedom in electromagnetic systems. In particular, metamaterials, which utilize engineered structures with minimum feature sizes that are comparable or even smaller than the wavelength of the electromagnetic waves, show remarkable capabilities for achieving manipulations of light in spatial dimensions that are not available with natural materials^10^. Very recently, active metamaterials have been proposed, where both spatial and temporal properties of engineered materials can be manipulated simultaneously^11^. These active metamaterials can exhibit exotic physical phenomena due to the spatiotemporal control of the structure. As an important example, photonic time crystal, as an analogy of its condensed-matter counterpart which exhibit new phases of many-body quantum matter, has been recently proposed. In a photonic time crystal, the refractive index is undergoing modulation that is periodic but abrupt, and such time crystal exhibits novel effects including band gaps in the momentum space and unusual topologically non-trivial phase^12^. Lately, the propagation of the electromagnetic waves in spatiotemporal photonic crystals are also studied^13^.

A Perspective in eLight by Yin et al.^14^ envisions the confluence of these two fields, i.e., Floquet engineering and metamaterials, that gives rise to the new area of Floquet metamaterials, with the aim to achieve non-trivial dynamic controls in electromagnetic and acoustic waves. They provide an introduction on the theoretical framework of Floquet physics, which have been widely used in photonic systems described with the periodic dependence in the time dimension, and has strong connection to previous works in quantum mechanics that analyzes Schrödinger equations with time dependent Hamiltonian. They then discuss time-interfaces as temporal meta-atoms, where Floquet engineering can be mapped into metamaterials, with meta-atoms being modulated at timescales shorter than the temporal periodicity of electromagnetic waves. Combining the building blocks of Floquet physics and metamaterials, the authors then highlight opportunities as well as experimental challenges in the emerging area of Floquet metamaterials. Certainly there are many exciting opportunities from the viewpoint of fundamental physics. Examples include the Floquet engineering in designing materials with temporal non-locality^15^ and many-body physics with photons using material nonlinearities^16^. On the other hand, from the experiment point of view, to demonstrate many of these effects require modulations that are both fast and strong, which represent a challenge especially in the optical frequency range.

The quest to control electromagnetic waves has been of central importance both for fundamental science and for practical engineering. Floquet metamaterials, the connection between Floquet engineering and metamaterials, offer great opportunities for exploring fundamental physics associated with non-locality, non-linearity, non-Hermiticity, non-reciprocity, and non-trivial topology, and may lead to new applications in controlling spatial, spectral, and temporal properties. One can therefore expect many more studies and researches in the field of Floquet metamaterials in the near future. The perspective of Yin et al.^14^ provides a timely review of this exciting emerging area.
